# Upregulation of the ErbB family by EZH2 in hepatocellular carcinoma confers resistance to FGFR inhibitor

**DOI:** 10.1007/s00432-021-03703-6

**Published:** 2021-06-22

**Authors:** Aldo Prawira, Thi Bich Uyen Le, Rebecca Zhi Wen Ho, Hung Huynh

**Affiliations:** Laboratory of Molecular Endocrinology, Division of Cellular and Molecular Research, National Cancer Centre Singapore, 11 Hospital Crescent, Singapore, 169610 Singapore

**Keywords:** Infigratinib, Varlitinib, Hepatocellular carcinoma, Liver cancer

## Abstract

**Purpose:**

Hepatocellular carcinoma (HCC), the most common manifestation of liver cancer, is one of the leading causes of cancer-related mortality worldwide with limited treatment options. Infigratinib, a pan-FGFR inhibitor, has shown a potent antitumour effect in HCC. However, drug resistance is often observed in long-term treatment. In this study, we examined the potential feedback mechanism(s) leading to infigratinib and explored a combination therapy to overcome resistance in HCC.

**Methods:**

Patient-derived xenograft (PDX) tumours were subcutaneously implanted into SCID mice and were subsequently treated with infigratinib. Tumour growth was monitored over time, and tumour samples were subjected to immunohistochemistry and Western blotting. For drug combination studies, mice were treated with infigratinib and/or varlitinib. Gene overexpression and knockdown studies were conducted to investigate the relationship between EZH2 and ErbB activity in infigratinib resistance.

**Results:**

Infigratinib-resistant tumours exhibited higher levels of p-ErbB2 and p-ErbB3, concomitant with an increase in EZH2 expression. Gene overexpression and knockdown studies revealed that EZH2 directly regulates the levels of p-ErbB2 and p-ErbB3 in acquired resistance to infigratinib. The addition of varlitinib effectively overcame infigratinib resistance and prolonged the antitumour response, with minimal toxicity.

**Conclusion:**

The upregulation of the ErbB family by EZH2 appears to contribute to infigratinib resistance. The combination of infigratinib and varlitinib showed a potent antitumour effect and did not result in additional toxicity, warranting further clinical investigation.

**Supplementary Information:**

The online version contains supplementary material available at 10.1007/s00432-021-03703-6.

## Introduction

Nearly 850,000 new cases of liver malignancy were diagnosed in 2018, constituting approximately 4.7% of all cancer cases. In the same year, close to 800,000 fatalities were reported, reflecting the high mortality rate of this disease (Ferlay et al. 2019). Pathologically, hepatocellular carcinoma (HCC) accounted for 85% of the total diagnoses. Since 2007, sorafenib has been the standard systemic therapy, despite its modest benefit of prolonging patients’ survival by around 3 months (Llovet et al. 2008). The recent approval for the combination of atezolizumab and bevacizumab represents a major landmark in HCC treatment (Cheng et al. 2019a; Finn et al. 2020). The drug combination conferred a significantly longer survival benefit and progression-free survival, with a 67.2% overall survival at 12 months, compared with 54.6% in sorafenib-treated patients (Finn et al. 2020). While the long-term prognosis of HCC treated with atezolizumab/bevacizumab is still unknown, the current overall 5-year survival of HCC patients receiving other therapies was estimated at less than 12% (Bray et al. 2018). This highlights the need to improve the treatment options for certain subsets of patients with oncogene-driven HCC.

Approximately 80% of HCC showed an aberrantly high expression of at least one fibroblast growth factor (FGF) and/or its receptor (FGFR) (Gauglhofer et al. 2011). The overexpressed FGF/FGFR have been reported to be involved in liver carcinogenesis (Wu et al. 2010), progression, and metastasis (Sandhu et al. 2014; Zheng et al. 2016) and thus, suggesting their potential as therapeutic targets. For example, the overexpression of FGFR-2 has been associated with a poorer cell differentiation, higher hepatic portal vein invasion, poorer prognosis, and tumour recurrence (Harimoto et al. 2010; Jun et al. 2020). The overexpression of FGFR3 was strongly correlated with a higher cancer stemness, higher nuclear grade, and angiogenesis-dependent metastasis (Qiu et al. 2005; Liu et al. 2016; Paur et al. 2020). Similarly, elevated levels of FGF19/FGFR4 signalling have been associated with a poorer prognosis (Kang et al. 2019), early tumour recurrence (Hyeon et al. 2013; Lin et al. 2019), and resistance to sorafenib (Gao et al. 2017).

The pan-FGFR inhibitor, infigratinib (NVP-BGJ398), is a selective small molecule with a sub-nanomolar affinity to FGFR1-4 (Guagnano et al. 2011, 2012). Several clinical trials on infigratinib for FGFR-driven cancers are ongoing. Notably, a phase II study in patients with advanced cholangiocarcinoma has reported clinically meaningful activity, with a manageable toxicity (Javle et al. 2018a, b). An earlier study on solid tumours harbouring FGFR alteration also suggested promising antitumour activity in FGFR1-amplified squamous cell non-small cell lung cancer and FGFR3-mutant urothelial cancer, while also showing a favourable safety profile (Nogova et al. 2017).

Previously, we have shown that infigratinib significantly inhibited tumour growth, reduced cell proliferation, normalised the intra-tumoral blood vessel, and impaired metastasis in HCC (Huynh et al. 2019). Moreover, infigratinib induced terminal differentiation in HCC cancer stem cells, thereby providing a longer lasting cytostatic effect (Prawira et al. 2020). However, we observed that approximately 20% of HCC PDX models did not respond to infigratinib, despite expressing FGFR1-4. In this study, we investigated the molecular mechanisms behind infigratinib-resistant HCC and explored the addition of varlitinib to overcome infigratinib resistance. Varlitinib is a pan-ERBB inhibitor that has been shown to suppress tumour growth via inhibition of the ERBB/ERK signalling (Liu et al. 2019). Clinical investigations on varlitinib are still in the early phase. However, the recently completed phase I study of varlitinib in ERBB2-positive advanced solid tumours has indicated a durable response with favourable safety profile, highlighting the potential for treatment with varlitinib (Tan et al. 2019).

## Materials and methods

Drug preparation and reagents used in this study are stated in the Supplementary Materials and Methods (see Supplementary Material).

### In vivo model

Male C.B-17 SCID mice aged 9–10 weeks, weighing 23–25 g (InVivos Pte. Ltd., Singapore), were provided with sterilised food and water and housed in negative pressure isolators set at 23 °C and 43% humidity, with 12-h light/dark cycles.

To assess the antitumour activity of infigratinib and/or varlitinib, previously established HCC PDX models (Huynh et al. 2006) expressing FGFR1–4 were subcutaneously implanted. The characteristics of the PDX models used in this study are listed in Supplementary Table 1. All treatments were initiated when the tumour size reached approximately 100–150 mm^3^, and mice were sacrificed when the tumour size reached approximately 2000 mm^3^. The mice were orally administered with vehicle, 15 mg/kg of infigratinib once daily, 50 mg/kg of varlitinib twice daily, or a combination of 15 mg/kg of infigratinib (once daily) and 50 mg/kg of varlitinib (twice daily).

To generate infigratinib-resistant PDX lines, mice bearing HCC21-0208, HCC06-0606, HCC01-0909, or HCC26-0808A tumours were repeatedly dosed with 15 mg/kg of infigratinib, until tumour progression was observed. Tumour tissue was then collected and re-implanted, and the drug administration was repeated.

For the gene overexpression/knockdown study, 5 × 10^6^ cells were injected subcutaneously into SCID mice in the presence of 20% Matrigel (Corning Inc., NY, USA).

### In vitro drug-resistant cells

A drug-resistant in vitro model was generated by treating cells derived from HCC21-0208 PDX with an incremental concentration of infigratinib, starting with 1 µM. Cells were constantly cultured in the presence of infigratinib, and the surviving cells at each concentration were allowed to grow to reach confluency. The cells were then passaged and grown at a higher concentration of infigratinib, until full resistance to 4 µM infigratinib was achieved.

### Stable EZH2 overexpression and knockdown cells

A full-length EZH2 gene was cloned into pLX302 (Addgene #25896). shRNA targeting EZH2 was cloned into pLKO.1 (Addgene #8453). shRNA targeting luciferase was cloned into pLKO.1 and served as the control plasmid. To generate stable gene overexpression/knockdown cell lines, lentivirus was generated by co-transfecting pMDLg/pRRE (Addgene #12251), pRSV-Rev (Addgene #12253), pMD2.G (Addgene #12259), and either one of the expression or knockdown vectors into LentiX-293 T cells (Takara Bio, Kusatsu, Shiga, Japan). LentiX-293 T cells were transfected for 72 h at 37 °C and lentivirus was subsequently purified by centrifugation at 200×*g* followed by supernatant filtration using 0.45 µm sterile filter. Purified lentivirus was then used to transduce HCC21-0208 cells derived from PDX tumours in the presence of 10 µg/mL of hexadimethrine bromide (polybrene) for 24 h at 37 °C, followed by selection pressure with 5 µg/mL of puromycin.

### Preparation of RNA for RNA-seq

Total RNA was extracted by homogenising frozen tissue from PDX tumours in TRIzol reagent (Thermo Fisher, Waltham, MA, USA), followed by overnight precipitation with isopropanol at -20 °C. The RNA pellet was washed with 75% cold ethanol and reconstituted in DEPC water. RNA library was prepared using the TruSeq Stranded Total RNA kit (with Ribozero depletion) (Illumina San Diego, CA, USA) and was subsequently sequenced on the Novaseq 6000 sequencing system (Illumina, San Diego, CA, USA).

### Colony formation assay and cell cycle analysis

The colony formation assay was performed by initially seeding 2000 cells in a 10 cm petri dish in triplicate. The cells were allowed to adhere overnight and were subsequently treated with 0.5 µM of infigratinib, 0.75 µM of varlitinib, or a combination of 0.25 µM of infigratinib and 0.375 µM of varlitinib. The cultures were left for 14 days and subsequently fixed and stained with 25% methanol and 0.5% crystal violet solution. Colonies with more than 50 cells were quantified.

For cell cycle analyses, cells were treated with the indicated concentration of infigratinib and/or varlitinib for 48 h and fixed in 70% cold ethanol overnight. The cells were then stained with the FxCycle PI/RNase staining solution (Thermo Fisher, Waltham, MA, USA), according to the manufacturer’s protocol. At least 100,000 cells/samples were analysed on the BD FACSCanto II flow cytometer (Becton Dickinson, Franklin Lakes, NJ, USA), and the percentage of cells in each phase was determined using FlowJo (v.10, Treestar, OR, USA).

### Immunohistochemistry

Tumour tissue were collected and fixed in 10% formalin overnight and subsequently embedded in paraffin blocks. Tissue Sects. (5 µm) were immunostained with antibodies against p-Histone H3 Ser10, cleaved PARP, CD31, p-ErbB2, and p-ErbB3, and visualised with SignalStain Boost IHC Detection Reagent (Cell Signaling Technology). Images were taken using an Olympus BX60 microscope (Olympus, Tokyo, Japan). The contrast and brightness of the images were uniformly adjusted for clarity.

### Vessel perfusion study

Each mouse bearing tumour xenografts (vehicle- or drug-treated) was intravenously injected with 100 mg of biotinylated *Lycopersicon esculentum* (tomato) lectin (VectorLabs #B-1175), prepared in 100 μl of 0.9% NaCl. The tumours were collected 10 min after the lectin perfusion and fixed in 10% formalin for paraffin embedding, before obtaining 5 μm sections. To visualize productive microvessels, immunohistochemistry was performed using the streptavidin–biotin peroxidase complex method (Lab Vision Corporation, Fremont, CA, USA). To quantify the mean microvessel density in the sections, 10 random 0.159 mm^2^ fields at a magnification of 100× were captured for each tumour.

For tumour hypoxia staining, the mice were intraperitoneally injected with 60 mg/kg of pimonidazole hydrochloride 1 h prior to the tumour collection. Tumour sections were then stained with the HypoxyProbe Plus Kit HP2 (HypoxyProbe Inc.) to identify hypoxic regions.

### Western blot analysis

The total protein was extracted by homogenising vehicle- or drug-treated tumours in a buffer containing 50 mM of Tris–HCl pH 7.4, 150 mM of NaCl, 0.5% NP-40, 1 mM of EDTA, and 25 mM of NaF, supplemented with proteinase inhibitors and 10 mM of Na_3_VO_4_. Eighty micrograms of protein were separated by SDS-PAGE and subsequently transferred onto a nitrocellulose membrane, followed by overnight incubation with primary antibodies at 4 °C. The membranes were then incubated with horseradish peroxidase-conjugated secondary antibodies for 1 h at room temperature, followed by visualisation using the enhanced chemiluminescent detection system (Amersham, Pharmacia Biotech).

### Statistical analyses

To compare the treatment groups, two-way ANOVA was performed. Student’s t test was used to compare the mean tumour weight at sacrifice and body weight at the end of the treatment cycle, and the number of colonies was determined using the colony formation assay. The Shapiro–Wilk normality test was conducted prior to the Student’s *t* test analysis. *p* ≤ 0.05 was considered statistically significant. Graphs were generated using GraphPad Prism 8 (v. 8.2.1).

## Results

### Infigratinib-resistant PDXs express higher levels of the ErbB family

**Fig. 1:**
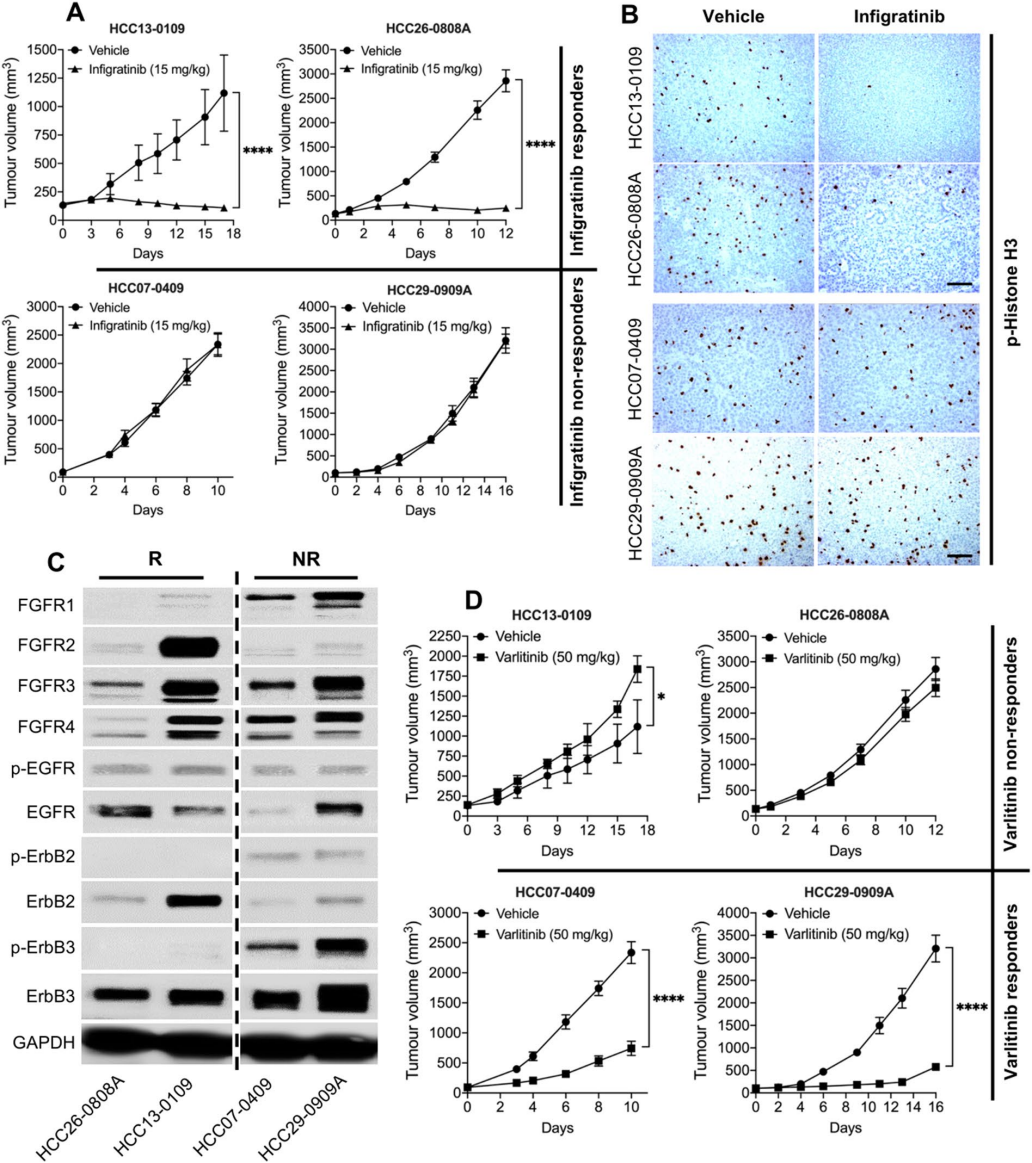
Infigratinib-resistant PDXs express higher levels of phosphorylated ErbB family proteins. HCC13-0109, HCC26-0808A, HCC07-0409, and HCC29-0909A tumours were subcutaneously implanted into SCID mice (*n* = 10 mice per group) and subsequently treated with 15 mg/kg of infigratinib once daily for the indicated time (**A**). Tissue sections from tumours obtained at the end of treatment cycle were stained for p-Histone H3 to examine proliferating cells (**B**). Western blot analyses showing the expression of FGFRs and ErbB receptors in responder and non-responder tumours. All four samples were run on the same gel but not consecutive lanes (**C**). The same tumours were subcutaneously implanted into SCID mice (*n* = 10 mice per group) and treated with 50 mg/kg of varlitinib twice daily for the indicated time (**D**). All mice treatments were initiated when the tumour volume reached approximately 100–150 mm^3^. The mean tumour volume ± SE is plotted. Scale bar: 100 µm. R, responders; NR, non-responders. *p < 0.05; ****p ≤ 0.0001

In our previous study, we showed that tumours expressing FGFR1-4 are sensitive to infigratinib (Huynh et al. 2019). Figure 1A shows that the tumour growth for HCC13-0109 and HCC26-0808A was significantly inhibited following treatment with infigratinib (*p* < 0.0001). However, HCC07-0409 and HCC29-0909A treated with infigratinib showed an almost identical growth pattern to those treated with the vehicle, suggesting that they are ‘non-responders’. Consistently, the ‘responder’ tumours (HCC26-0808A and HCC13-0109) showed a significantly lower number of p-Histone H3, while non-responder tumours did not show a significant difference in the number of cells staining for p-Histone H3 (Fig. 1B). The infigratinib responders displayed an increase in the number of cleaved PARP-positive cells and a more ‘normalised’ blood vessel organisation, compared with those of non-responders (Supplementary Fig. 1). The apparent differences between the responders and non-responders were observed, despite all the PDX lines expressing measurable levels of FGFR-2, -3, and/or -4 (Fig. 1C). The infigratinib non-responders expressed even higher levels of FGFR1 than infigratinib responders. This suggests that there is a mechanism of inherent resistance to FGFR inhibitor.

Western blot analyses indicated that the non-responders express higher levels of the phosphorylated ErbB family (Fig. 1C). In particular, they express higher levels of p-ErbB2 and p-ErbB3, which led us to hypothesise that those infigratinib-resistant PDXs would respond to the pan-ErbB inhibitor, varlitinib. Indeed, Fig. 1D showed that the growth of HCC07-0409 and HCC29-0909A tumours was significantly inhibited by varlitinib. In contrast, HCC13-0109 and HCC26-0808A, which did not express detectable levels of p-ErbB2 and p-ErbB3, did not show growth inhibition, following varlitinib treatment. These results suggests that the ErbB family of proteins might play a role in infigratinib resistance and that varlitinib would be more effective in FGFR-driven HCC, in which higher levels of p-ErbB2 and/or p-ErbB3 are expressed.

**Fig. 2:**
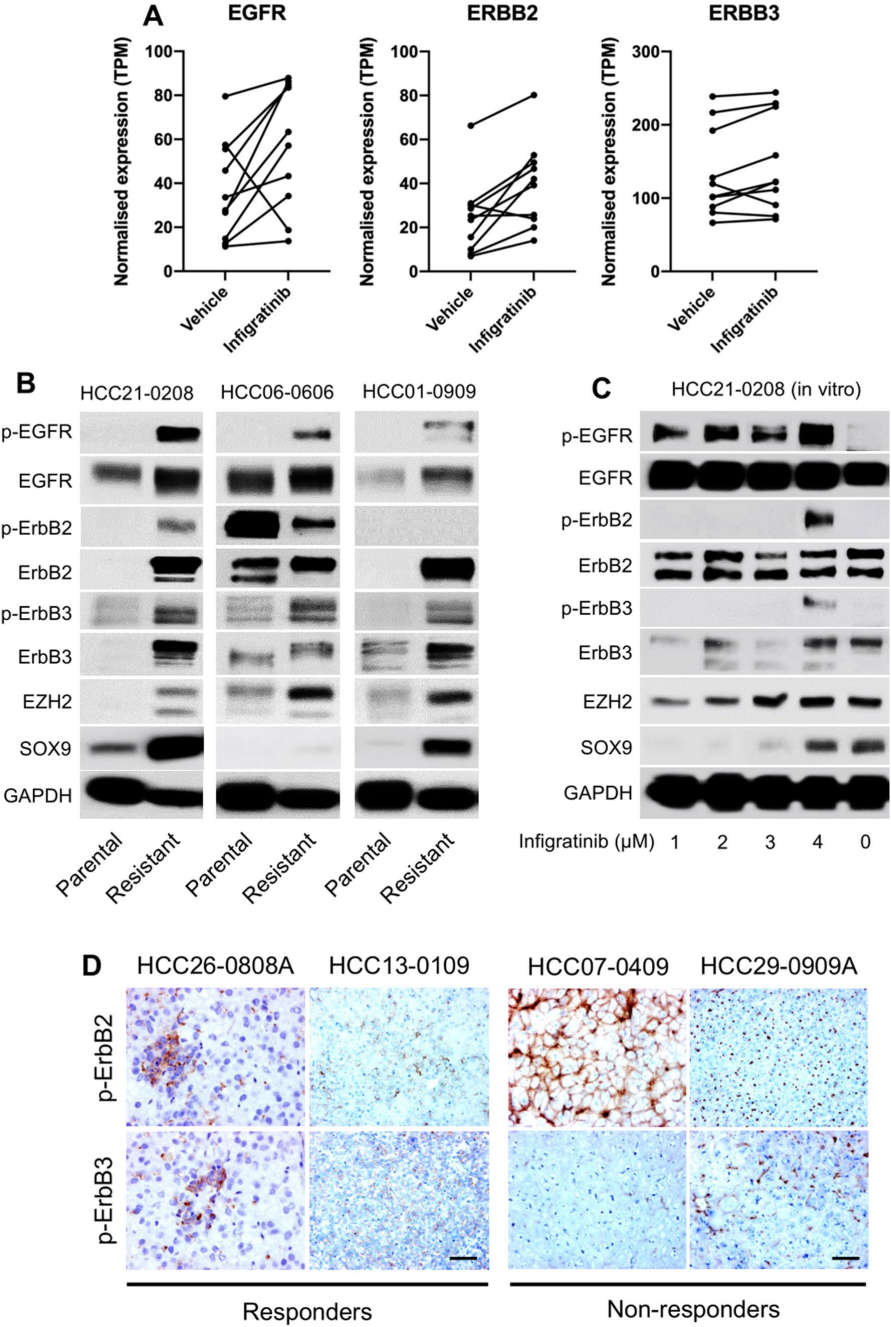
Treatment with infigratinib increases the expression of ErbB family proteins. RNA-seq analysis for the expression of EGFR, ErbB2, and ErbB3 from 10 PDX tumours treated with infigratinib (**A**). Tumour samples from previously established infigratinib-resistant HCC21-0208, HCC06-0606, and HCC01-0909 were subjected to Western blot analyses (**B**). Infigratinib-resistant HCC21-0208 were generated by treating cells with an increasing concentration of infigratinib, and the total protein extracted from each stage of resistance was subjected to Western blot analyses. Representative blots are shown (**C**). Immunohistochemical analyses of p-ErbB2 and p-ErbB3 in responder and non-responder tumours, following infigratinib treatment, were conducted. Representative images are shown (**D**)

To establish its involvement in infigratinib resistance, we investigated whether the ErbB family was upregulated following infigratinib treatment. We analysed the RNA-seq data from 10 PDXs, which initially showed a response to infigratinib. The total mRNA from the tumour samples collected at the end of treatment cycle (between 18 and 32 days) was subjected to an RNA-seq. Generally, an upward trend was observed in the mRNA levels of total EGFR, ErbB2, and ErbB3 from the PDX tumours treated with infigratinib (Fig. 2A). This was supported by our previously established infigratinib-resistant PDX models. Western blot analyses showed that infigratinib-resistant HCC21-0208, HCC06-0606, and HCC01-0909 tumours constitutively express higher levels of p-EGFR, p-ErbB2, and p-ErbB3 (Fig. 2B). This suggests that the upregulation of the ErbB family, at least in tumours with acquired resistance, is the result of an adaptive response to infigratinib treatment.

Furthermore, we established infigratinib-resistant HCC21-0208 cells in vitro by culturing cells for several passages in an increasing concentration of infigratinib. Similarly, long-term treatment with infigratinib resulted in a higher expression of p-EGFR, p-ErbB2, and p-ErbB3 (Fig. 2C). We also observed an increase in the markers of cell stemness, such as EZH2 and SOX9 (Fig. 2B, C). Initially, treatment with infigratinib significantly reduced the levels of EZH2 and SOX9. However, the cells appeared to regain the expression of these stem cell markers as they became more resistant to infigratinib (Fig. 2C).

Immunohistochemical analyses of early infigratinib-resistant HCC13-0109 and HCC26-0808A revealed clusters of cells expressing p-ErbB2 and p-ErbB3. Staining was specific to these nodules, as indicated by the absence of staining-positive cells in other areas of tissue sections. Notably, these cells were more tightly clustered, resembling undifferentiated cells. Contrarily, non-responder tumours (HCC07-0409 and HCC29-0909A) showed large areas that are positive for p-ErbB2 or p-ErbB3 (Fig. 2D), which is consistent with previous Western blot analyses (Fig. 1C).

### EZH2 regulates the expression of ErbB2 and ErbB3

**Fig. 3:**
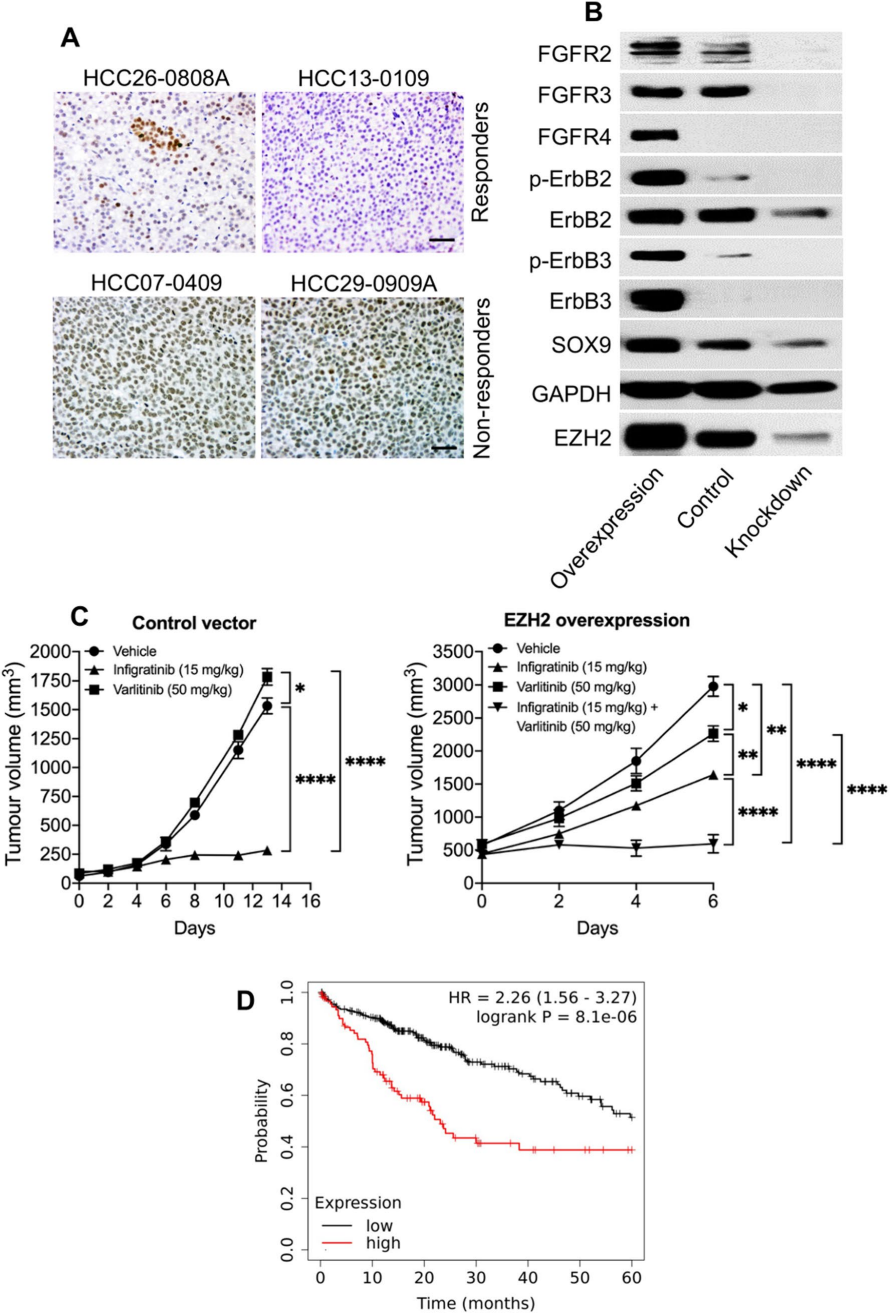
EZH2 regulates the expression of ErbB family proteins. Tissue sections from infigratinib responder and non-responder tumours were stained for EZH2 (**A**). HCC21-0208 cells with a stable EZH2 overexpression and knockdown were subcutaneously implanted into SCID mice. Protein samples were extracted from tumour tissue and subjected to Western blot analyses (**B**). HCC21-0208 tumours with EZH2 overexpression and a control were treated with 15 mg/kg of infigratinib and/or 50 mg/kg of varlitinib. The mean tumour volume ± SE was compared using two-way ANOVA (**C**). Kaplan–Meier analysis showing the five-year overall survival of HCC patients expressing high and low levels of EZH2 (**D**). *p < 0.05; ***p ≤ 0.001; ****p ≤ 0.0001

Given that our results suggested an association between the levels of EZH2 and the expression of ErbB proteins in infigratinib-resistant tumours, we hypothesised that EZH2 plays a role in the regulation of FGFR/ErbB proteins. Immunohistochemical staining indicated that infigratinib-treated tumours of responders were largely negative for EZH2. Small clusters of cells that were positively stained for EZH2 were detected at the end of treatment cycle, while non-responders showed EZH2-positive cells in large sections of the tumour (Fig. 3A). This staining pattern is consistent with the expression pattern of p-ErbB2 and p-ErbB3 (Fig. 2D).

Western blot analysis suggested that the overexpression of EZH2 increased the levels of ErbB2, p-ErbB2, ErbB3, p-ErbB3, FGFR2-4, and the stem cell marker, SOX9. Conversely, the knockdown of EZH2 abolished the expression of these proteins (Fig. 3B). These data suggest that EZH2 directly plays a role in the regulation of FGFR/ErbB proteins. We then investigated whether EZH2-overexpressing tumours are more resistant to infigratinib, and whether they are more sensitive to varlitinib. In HCC21-0208 tumour transduced with a control vector, infigratinib potently inhibited the tumour growth. While infigratinib suppressed the growth of the HCC21-0208 tumour overexpressing EZH2, the tumour inhibition was less than that of the control vector tumour, as suggested by the difference in tumour burden over time (Fig. 3C). Varlitinib did not suppress the growth of tumours transduced with a control vector. Conversely, HCC21-0208 overexpressing EZH2 was more sensitive to varlitinib. However, the tumour inhibition by infigratinib and varlitinib in EZH2-overexpressing HCC21-0208 was not complete, which was presumably due to the activation of both the FGFR and ErbB pathways. The co-administration of varlitinib and infigratinib suppressed tumour growth more effectively, compared with varlitinib or infigratinib alone (Fig. 3C).

Furthermore, we analysed the correlation between the levels of EZH2 and the overall survival of HCC patients. Survival analysis of 322 patients from the Kaplan–Meier Plotter database (https://kmplot.com/analysis/) showed that patients with a high EZH2 had a significantly lower overall survival rate, compared to those with a low expression of EZH2 (Fig. 3D).

### Varlitinib overcomes resistance to infigratinib

**Fig. 4:**
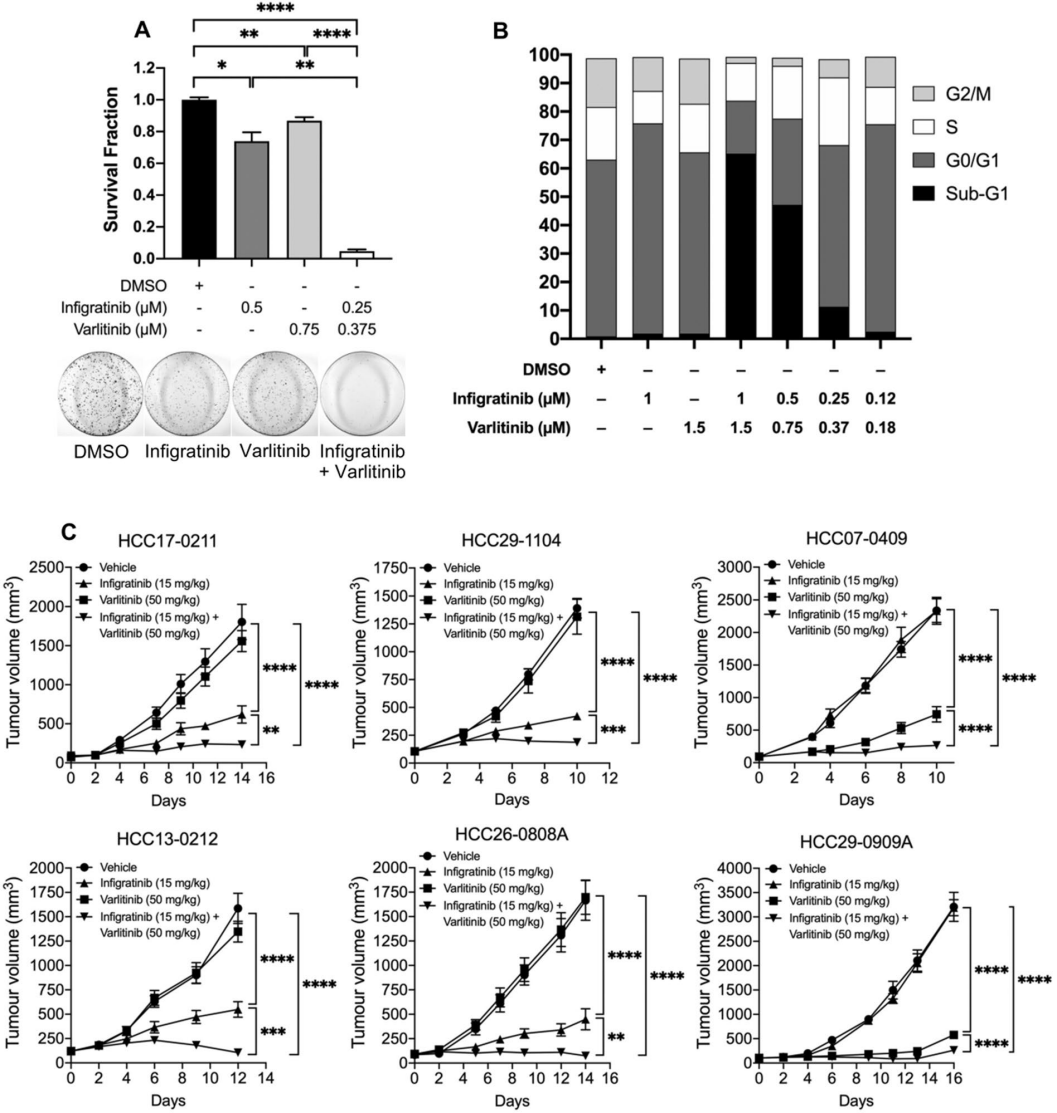
Varlitinib reverses infigratinib resistance. HCC21-0208 cells were treated with the indicated concentration of infigratinib and/or varlitinib and allowed to form colonies. Colonies of more than 50 cells were quantified, and the mean colony number ± SE from three independent experiments was compared using Student’s *t* test (**A**). HCC21-0208 cells were treated with the indicated concentrations of infigratinib and/or varlitinib and subjected to cell cycle analyses (**B**). The indicated tumours were subcutaneously implanted into SCID mice (*n* = 10 mice per group) and subsequently treated with 15 mg/kg of infigratinib and/or 50 mg/kg of varlitinib (**C**). The mean tumour volume ± SE was compared using two-way ANOVA. *p < 0.05; **p ≤ 0.01; ***p ≤ 0.001; ****p ≤ 0.0001

Since the ErbB family appears to play a role in adaptive resistance to infigratinib, we sought to determine whether the addition of the pan-ErbB inhibitor, varlitinib, could reverse resistance and prolong the antitumour effect of infigratinib. A colony formation assay showed that treatment with infigratinib or varlitinib alone reduced the number of colonies by approximately 30% and 10%, respectively, compared with the vehicle treatment. However, when combined, infigratinib and varlitinib significantly reduced the number of colonies by more than 90% (Fig. 4A). Cell cycle analyses suggests that the antitumour effect of infigratinib and varlitinib combined is achieved primarily due to the induction of apoptosis, as indicated by the increase in the proportion of cells at the sub-G1 phase (Fig. 4B).

We then investigated whether the antitumour effect of infigratinib and varlitinib combined can be achieved in vivo. Figure 4C suggests that in the FGFR-dependent HCC17-0211, HCC29-1104, HCC13-0212, and HCC26-0808A, treatment with varlitinib did not appear to show an antitumour effect. In contrast, infigratinib significantly suppressed tumour growth. However, we observed that all tumours treated with infigratinib alone progressed over time. The combination of varlitinib and infigratinib significantly inhibited tumour growth, with no signs of tumour progression until the end of treatment cycle (Fig. 4C). Similarly, in the ErbB-dependent HCC07-0409 and HCC29-0909A, the combination of varlitinib and infigratinib potently impaired tumour growth, compared with varlitinib treatment alone. This suggests that the dual inhibition of the FGFR and ErbB pathways provides a superior antitumour effect than treatment with infigratinib or varlitinib alone. Importantly, we did not observe signs of clinical toxicity, such as a significant body weight loss and reduced grooming frequency, motor activity, food, and/or water consumption (Supplementary Fig. 2).

**Fig. 5:**
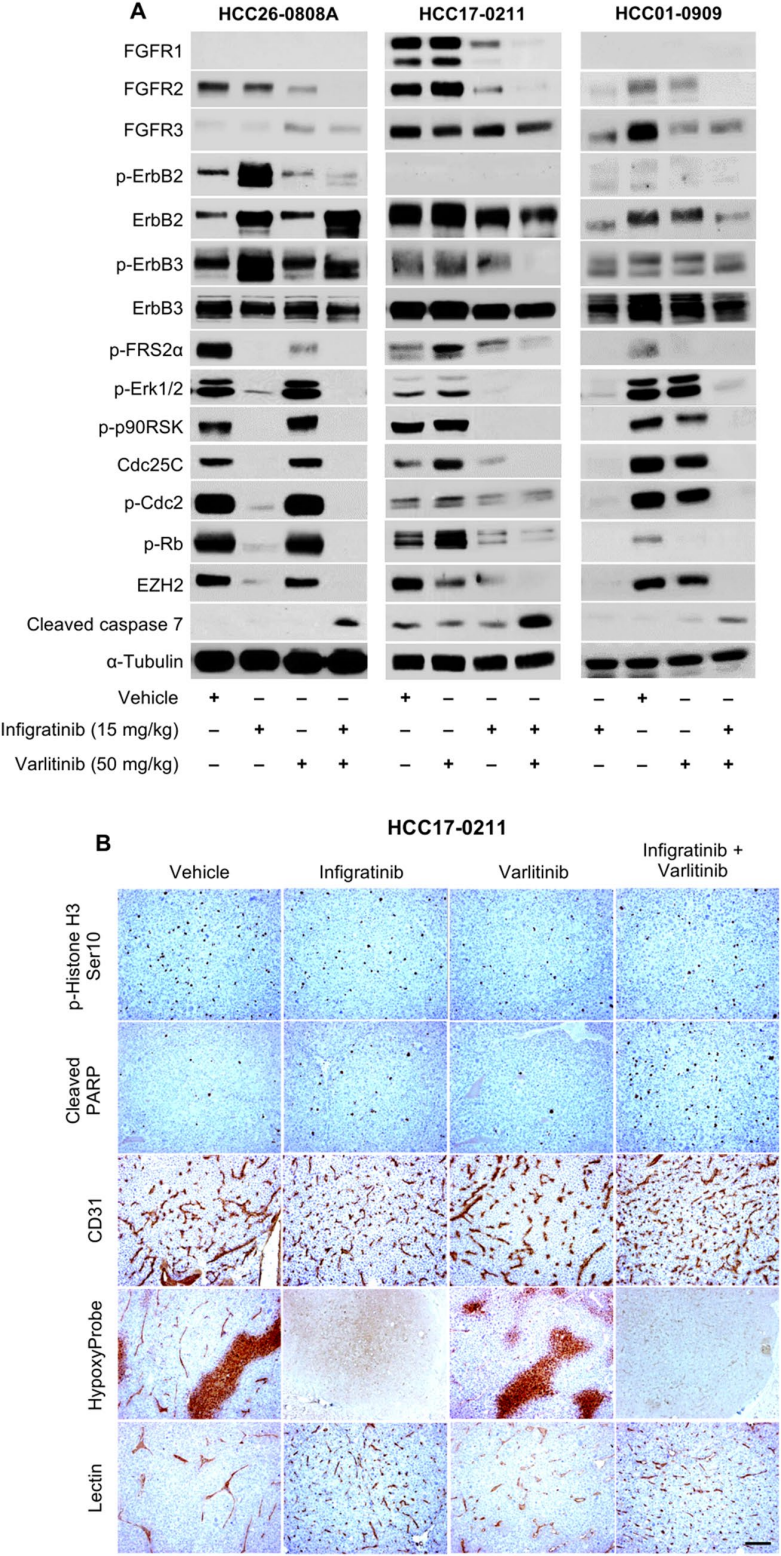
The effects of varlitinib and infigratinib on signalling proteins, cell proliferation, hypoxia, and intra-tumoral blood vessels. Mice bearing HCC26-0808A, HCC17-0211, and HCC01-0909 tumours were treated for 14 days, and the tumour lysate was subjected to Western blot analyses. The treatments were initiated when the tumours reached approximately 100–150 mm^3^. The membranes were incubated with the indicated antibodies, and representative blots are shown (**A**). Tissue sections from the HCC17-0211 tumour were stained for p-Histone H3, cleaved PARP, CD31, and hypoxia (HypoxyProbe) (**B**)

Further investigation on the molecular mechanism of infigratinib and varlitinib combined revealed an inhibition of both the FGFR and ErbB receptors. This, in turn, abolished the expression of proteins involved in signal transduction, proliferation, and cell cycle, such as p-FRS2α, p-Erk1/2, p-p90RSK, Cdc25C, p-Cdc2, and p-Rb. In addition, the drug combination also increased the expression of cleaved caspase 7, indicating an increase in apoptotic cells (Fig. 5A), which is consistent with our previous cell cycle analyses (Fig. 4B).

Moreover, immunohistochemical analyses indicated a significant decrease in the levels of p-Histone H3 and an increase in cleaved PARP. CD31 staining of the HCC17-0211 tumour treated with infigratinib and the combination of infigratinib and varlitinib showed evidence of intra-tumoral blood vessel remodelling, where blood vessels appear more ‘normalised’, with capillary-like structures, compared with the dysregulated blood vessels in the vehicle and varlitinib-treated tumours. The lectin perfusion study suggested that the normalised blood vessels are mostly functional. Moreover, the normalised blood vessels are associated with a significant reduction in the hypoxic region, as indicated by HypoxyProbe staining (Fig. 5B). Similar data were obtained when the HCC26-0808A and HCC01-0909 samples were analysed.

## Discussion

To date, the incidence and mortality of HCC has continually risen, particularly in Western countries, while it has remained high in Asia (Dasgupta et al. 2020). A recent IMbrave150 study demonstrated a promising outcome of atezolizumab and bevacizumab in improving the overall survival and progression-free survival, compared with sorafenib (Cheng et al. 2019b; Finn et al. 2020). However, a considerable proportion of patients still do not respond to immune checkpoint inhibitors. Since approximately 80% of HCC showed an aberrantly high expression of FGF/FGFR, it is still necessary to improve the treatment in oncogene-driven HCC. Our previous studies have shown the antitumour potential of inhibiting the FGF/FGFR pathway using infigratinib alone or in combination with other drugs (Huynh et al. 2019; Prawira et al. 2020). However, drug resistance often develops, rendering the treatment ineffective. Here, we utilised PDX models to explore one of the pathways involved in infigratinib resistance and provided evidence to overcome drug resistance in HCC.

In the first part of the study, we reported that a small proportion of PDX lines did not respond to infigratinib, despite expressing FGFRs. These lines exhibited higher levels of p-ErbB2 and p-ErbB3, which led us to hypothesise that these PDX models have switched their growth dependence to the ErbB pathway. Indeed, the inhibition of the ErbB pathway with varlitinib significantly suppressed the growth in these models (Fig. 1A–D). Since a higher expression of phosphorylated ErbB appeared to play a role in infigratinib resistance, we hypothesised that those PDX models that acquired resistance to infigratinib also activated the ErbB pathway. Our hypothesis was supported by RNA-seq analysis, showing that following long-term treatment with infigratinib, the levels of EGFR, ErbB2, and ErbB3 were increased. This was also observed in our in vitro infigratinib-resistant HCC21-0208 cells (Fig. 2A–C). Consistently, a previous study on the FGFR3-dependent RT112 cell line showed that treatment with infigratinib increased the expression of the ErbB family and shifted its growth dependence to the ErbB pathway (Wang et al. 2015). Similarly, infigratinib-resistant breast cancer was also reported to exhibit elevated levels of EGFR (Holdman et al. 2015).

Next, we sought to determine the pathways involved in FGFR/ErbB regulation. We observed that the levels of EZH2 were higher in infigratinib-resistant PDX models. Moreover, the EZH2 levels appeared to increase as the cells became more resistant to infigratinib, and this was associated with an increase in p-EGFR, p-ErbB2, and p-ErbB3. Immunohistochemical staining indicated that PDX tumours that did not respond to infigratinib highly and uniformly express EZH2 (Fig. 3A), p-ErbB2, and/or p-ErbB3 (Fig. 2D). Interestingly, we detected small clusters of cells that are positive for EZH2 in infigratinib responders after long-term treatment with infigratinib (Fig. 3A). These cells have a similar expression pattern as p-ErbB2 and p-ErbB3 (Fig. 2D) and were presumably present at the early stage of acquiring resistance.

The overexpression of EZH2 appeared to increase the levels of p-ErbB2 and p-ErbB3, while the knockdown of EZH2 reduced the expression of these proteins (Fig. 3B), suggesting that EZH2 upstream of ErbB2/3 indeed plays a role in the regulation of the ErbB family of proteins. Furthermore, PDX tumours overexpressing EZH2 appeared to gain resistance to infigratinib and showed a better response to varlitinib (Fig. 3C). However, the combined inhibition of ErbB and FGFR is necessary to completely inhibit tumour growth, suggesting that there is some level of dynamic dependence to both ErbB and FGFR in infigratinib-resistant tumours. Indeed, we observe this in other PDX lines, where the inhibition of both ErbB and FGFR abolished the EZH2 expression and provided a longer lasting tumour suppression (Fig. 4C). Previous studies have demonstrated the involvement of EZH2 in HCC. For example, aberrantly high levels of EZH2 have been reported to be involved in hepatocarcinogenesis (Xu et al. 2020) and correlated with a poorer overall survival, progression-free survival, and relapse-free survival (Guo et al. 2020), which is consistent with our survival analysis. Here, we reported the correlation between the levels of EZH2, p-ErbB2, and p-ErbB3, which could potentially be used as a predictive biomarker for infigratinib or varlitinib treatment.

Mechanistically, infigratinib and varlitinib combined abrogated the expression of FGFRs and phosphorylated ErbB, which, in turn, attenuated the proteins involved in cell cycle, proliferation, and growth, including p-Erk1/2, p-p90RSK, p-Cdc2, Cdc25C, and p-Rb. Moreover, the drug combination increased the proportion of apoptotic cells, as indicated by the apoptotic markers, cleaved caspase 7 and cleaved PARP. Infigratinib and varlitinib also significantly reduced hypoxia and appeared to normalise blood vessels, while increasing functional blood vessels (Fig. 5B). This was consistent with our previous report on the effects of infigratinib on blood vessel normalisation and intra-tumoral hypoxia (Huynh et al. 2019).

**Fig. 6:**
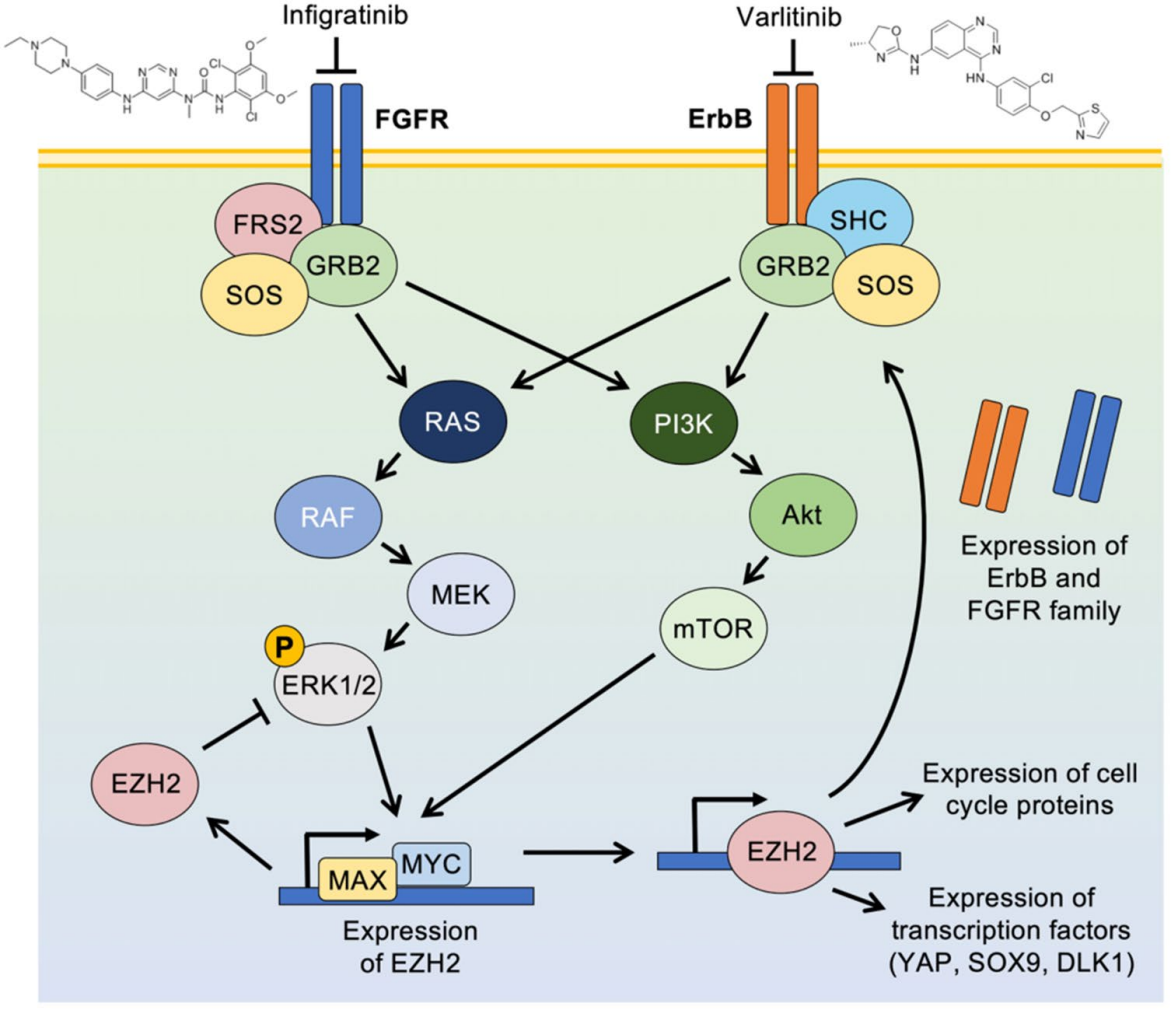
Proposed mechanism of FGFR/ErbB regulation by EZH2. The initial inhibition of FGFR by infigratinib leads to the downregulation of its downstream signalling proteins and several stem cell markers, including EZH2. However, long-term treatment eventually reactivates EZH2, which leads to an increase in the expression of the ErbB family and FGFRs. The dual inhibition of FGFR and ErbB effectively blocks resistance and results in a longer antitumour effect

Taken together, these data suggest the progressive events leading to infigratinib resistance. Specifically, the initial inhibition of the FGFR pathway leads to the downregulation of markers of cell stemness, including EZH2 and SOX9. However, as cells gain resistance, there is a re-expression in the levels of these markers, which was associated with an increase in the ErbB family proteins and its phosphorylated forms. This is evident from the formation of resistant nodules that constitutively express p-ErbB2 and p-ErbB3. Consequently, activated ErbB proteins confer resistance to infigratinib by switching the growth dependence to the EZH2/ErbB pathway, allowing resistant nodules to grow and form the bulk of the tumour (Fig. 6). While treating infigratinib-resistant tumours with varlitinib provides a minor antitumour effect, it appears that complete inhibition is only achieved by the dual inhibition of FGFR and ErbB. Clinically, infigratinib has shown promising activity in several clinical trials, including trials for advanced cholangiocarcinoma (Javle et al. 2018a, b) and advanced urothelial carcinoma (Pal et al. 2018). While varlitinib has not been extensively studied clinically, a small clinical trial on advanced solid tumours has indicated that it is well tolerated and showed a durable response in HER2-positive ovarial clear cell cancer (Tan et al. 2019). Given the high efficacy of infigratinib and varlitinib combined and the favourable safety profile in our preclinical study, determining the clinical efficacy of the combined treatment in patients with FGFR/ErbB-dependent tumours is warranted.

## Supplementary Information

Below is the link to the electronic supplementary material.

Supplementary file1 (DOCX 17 KB)

Supplementary file2 (DOCX 17 KB)

Supplementary file3 (TIFF 1576 KB) Supplementary Figure 1. Immunohistochemical staining for cleaved PARP and CD31 in responders and non-responders. Mice bearing the indicated tumours were treated with 15 mg/kg of infigratinib once daily. Tissue sections from the tumours collected at the end of treatment cycle were stained for cleaved PARP and CD31 (blood vessels). Responder tumours showed higher levels of cleaved PARP and a more ‘normalised’ intra-tumoral blood vessel structure. Non-responders did not show a significant increase in cleaved PARP-positive cells and exhibited an unregulated blood vessel structure. Five images from random fields were captured using an Olympus BX60 microscope, with ×100 magnification. Representative images are shown.

Supplementary file4 (TIFF 743 KB) Supplementary Figure 2. Body weights of the mice treated with infigratinib and/or varlitinib. Mice bearing the indicated tumours (n = 10 mice per group) were treated with 200 µL of a vehicle, 15 mg/kg of infigratinib once daily, 50 mg/kg of varlitinib twice daily, or a combination of infigratinib and varlitinib. The body weight was monitored daily, and the mean body weight at the end of the treatment ± SE was plotted (A). Representative images of the mice treated with infigratinib and/or varlitinib are shown (B). The mice did not show significant signs of toxicity, as indicated by the body weight, motor activity, and well-groomed fur.

## Data Availability

The data that support the findings of this study are available on request from the corresponding author. The data are not publicly available due to privacy or ethical restrictions.

## Abbreviations

EGFR: Epidermal growth factor receptor
ERBB: ErbB family of receptor tyrosine kinases
EZH2: Enhancer of zeste homolog 2
FGFR: Fibroblast growth factor receptor
HCC: Hepatocellular carcinoma
PDX: Patient-derived xenograft
SCID: Severe combined immunodeficient
SOX9: SRY-box transcription factor 9
