# Supplementary material for: Upregulation of the ErbB family by EZH2 in hepatocellular carcinoma confers resistance to FGFR inhibitor
Source: J Cancer Res Clin Oncol. 2021 Jun 22;147(10):2955–68. doi: 10.1007/s00432-021-03703-6 (PMC8397639; doi:10.1007/s00432-021-03703-6)
Supplement: Supplementary file 1 — Supplementary file1 (DOCX 17 KB) [file 432_2021_3703_MOESM1_ESM.docx]

**Supplementary Materials and Methods**

**Reagents**

The pan-FGFR inhibitor, infigratinib (S2183), and the pan-ERBB inhibitor, varlitinib (S2755), were purchased from Selleck Chemicals (Houston, TX, USA) and were dissolved in 21% Captisol and 30% PEG300 solution (vehicle) for oral administration. Antibodies against FGFR1 (#9740), FGFR3 (#4574), FGFR4 (#8562), p-EGFR Tyr1068 (#3777), EGFR (#4267), p-ErbB2 Tyr1221/1222 (#2243), ErbB2 (#4290), p-ErbB3 Tyr1289 (#4791), ErbB3 (#12708), EZH2 (#5246), SOX9 (#82630), p-FRS2α Tyr436 (#3861), p-Erk1/2 Thr202/Tyr204 (#4370), p-p90RSK Thr359/Ser363 (#9344), Cdc25C (#4688), p-Cdc2 Tyr15 (#9111), Cleaved PARP (#5625), cleaved caspase 7 (#9491), p-Rb Ser807/811 (#9308), α-tubulin (#2144), and CD31 (#77699) were obtained from Cell Signalling Technology (Beverly, MA, USA). Antibodies against FGFR2 (#sc-122) and GAPDH (#sc-166545) were purchased from Santa Cruz Biotechnology Inc. (Santa Cruz, CA, USA). Biotinylated *Lycopersicon esculentum* (tomato) lectin (#B-1175) was purchased from Vector Laboratories, Inc. (Burlingame, CA, USA). Pimonidazole hydrochloride and Hypoxyprobe plus Kit HP2 were obtained from Hypoxyprobe Inc. (Burlington, MA, USA).
