# Supplementary material for: Upregulation of the ErbB family by EZH2 in hepatocellular carcinoma confers resistance to FGFR inhibitor
Source: J Cancer Res Clin Oncol. 2021 Jun 22;147(10):2955–68. doi: 10.1007/s00432-021-03703-6 (PMC8397639; doi:10.1007/s00432-021-03703-6)
Supplement: Supplementary file 2 — Supplementary file2 (DOCX 17 KB) [file 432_2021_3703_MOESM2_ESM.docx]

**Supplementary Table 1.** The expression of FGFR1-4 in the PDX lines used in this study.

| **PDX line** | **FGFR1** | **FGFR2** | **FGFR3** | **FGFR4** |
| --- | --- | --- | --- | --- |
| HCC13-0109 | Low | High | High | High |
| HCC26-0808A | Undetectable | Low | High | High |
| HCC07-0409 | High | Low | High | High |
| HCC29-0909A | High | Low | High | High |
| HCC21-0208 | Undetectable | High | High | High |
| HCC06-0606 | Undetectable | High | High | High |
| HCC01-0909 | Low | High | High | High |
| HCC17-0211 | Undetectable | Intermediate | High | High |
| HCC13-0212 | Low | Intermediate | Intermediate | High |
| HCC29-1104 | Undetectable | Intermediate | High | High |
