# Supplementary figures and images for: Upregulation of the ErbB family by EZH2 in hepatocellular carcinoma confers resistance to FGFR inhibitor
Source: J Cancer Res Clin Oncol. 2021 Jun 22;147(10):2955–68. doi: 10.1007/s00432-021-03703-6 (PMC8397639; doi:10.1007/s00432-021-03703-6)

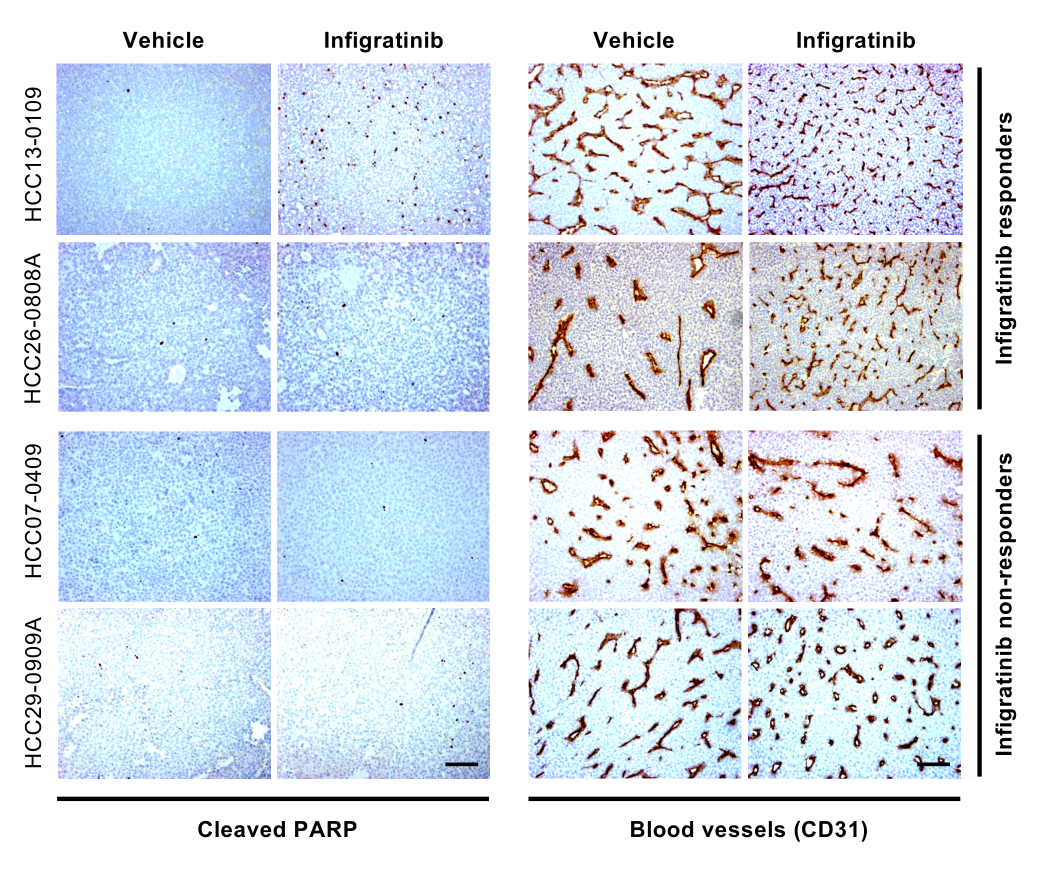

Supplement: Supplementary file 3 — Supplementary file3 (TIFF 1576 KB) Supplementary Figure 1. Immunohistochemical staining for cleaved PARP and CD31 in responders and non-responders. Mice bearing the indicated tumours were treated with 15 mg/kg of infigratinib once daily. Tissue sections from the tumours collected at the end of treatment cycle were stained for cleaved PARP and CD31 (blood vessels). Responder tumours showed higher levels of cleaved PARP and a more ‘normalised’ intra-tumoral blood vessel structure. Non-responders did not show a significant increase in cleaved PARP-positive cells and exhibited an unregulated blood vessel structure. Five images from random fields were captured using an Olympus BX60 microscope, with ×100 magnification. Representative images are shown. [file 432_2021_3703_MOESM3_ESM.tiff]

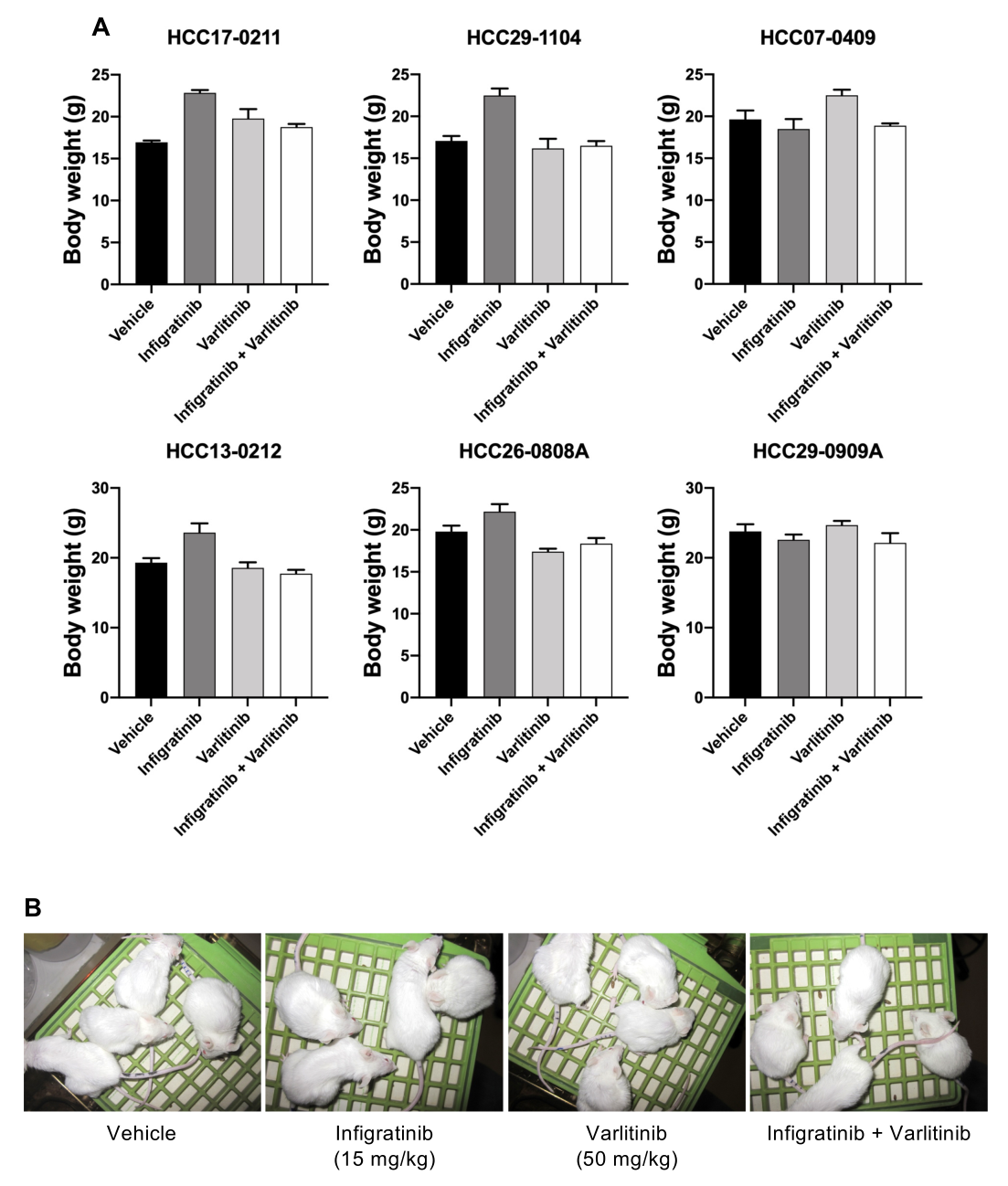

Supplement: Supplementary file 4 — Supplementary file4 (TIFF 743 KB) Supplementary Figure 2. Body weights of the mice treated with infigratinib and/or varlitinib. Mice bearing the indicated tumours (n = 10 mice per group) were treated with 200 µL of a vehicle, 15 mg/kg of infigratinib once daily, 50 mg/kg of varlitinib twice daily, or a combination of infigratinib and varlitinib. The body weight was monitored daily, and the mean body weight at the end of the treatment ± SE was plotted (A). Representative images of the mice treated with infigratinib and/or varlitinib are shown (B). The mice did not show significant signs of toxicity, as indicated by the body weight, motor activity, and well-groomed fur. [file 432_2021_3703_MOESM4_ESM.tiff]
